# Overcoming Data Scarcity: Few-Shot Pig Vocalization Recognition via Domain Expansion, Knowledge Transfer, and Feature Alignment

**DOI:** 10.3390/ani16132074

**Published:** 2026-07-05

**Authors:** Guangbo Li, Wenxiu Liu

**Affiliations:** 1 College of Electronic and Information Engineering, Huaibei Institute of Technology, Huaibei 235000, China; liguangbo@hblgxy.edu.cn; 2 College of Command and Control Engineering, Army Engineering University of the People’s Liberation Army, Nanjing 210007, China; 3 School of Information and Artificial Intelligence, Anhui Agricultural University, Hefei 230036, China

**Keywords:** pig vocalization, few-shot learning, bioacoustics, spectrogram classification, SpecAugment, ArcFace, self-supervised audio representation

## Abstract

Pig vocalization recognition can support non-invasive monitoring in precision livestock farming, but labelled pig-sound recordings are often limited for specific behaviours or physiological states. Under few-shot conditions, deep models may overfit, whereas traditional acoustic features may not fully describe class-specific time-frequency patterns. This study proposed PSA-AP, a pig-sound adaptation pipeline that uses log-Mel spectrograms and integrates SpecAugment-based domain expansion, ImageNet-pretrained ResNet18 knowledge transfer, and ArcFace-based feature alignment. The method was designed to reduce dependence on limited labelled samples, improve task-adapted representation learning, and enhance inter-class separability in the embedding space. Experiments were conducted on a five-class few-shot pig vocalization classification task, including eat, estrous, farrowing (fap), howl, and oink sounds collected from 10 adult Landrace pigs. Using K={5,10,15,20,25,30} labelled wav files per class and five random seeds, each selected training wav file and each held-out test wav file was converted into one 1.0 s log-Mel spectrogram for model training or evaluation. Final evaluation was based on the last checkpoint of each training run. PSA-AP achieved the best mean Accuracy, Macro-F1, and UAR at every K-shot setting. At K=30, PSA-AP reached 90.60% Accuracy, 90.49% Macro-F1, and 90.60% UAR, exceeding Raw by 7.80, 7.82, and 7.80 percentage points, respectively. These results indicate that the proposed integration of domain expansion, knowledge transfer, and feature alignment provides a feasible supervised adaptation strategy for few-shot pig vocalization recognition within the current protocol.

## Simple Summary

Pig vocal sounds can help monitor animal state without disturbing the animals, but labelled recordings for specific behaviours or physiological states are often scarce. This study focused on few-shot recognition of five pig vocalization categories: eat, estrous, farrowing (fap), howl, and oink. We proposed PSA-AP, a pig-sound adaptation pipeline that converts audio into log-Mel spectrograms and combines spectrogram augmentation, knowledge transfer, and feature alignment. Using the last training checkpoint as the final evaluation model, PSA-AP achieved the best mean Accuracy, Macro-F1, and UAR across all six K-shot settings from 5 to shot to 30-shot. At 30-shot, PSA-AP reached 90.60% Accuracy, 90.49% Macro-F1, and 90.60% UAR. These findings suggest that the proposed task-adapted supervised model is feasible and effective for limited-label pig-sound recognition under the current protocol.

## 1. Introduction

Pig vocalizations provide an important non-invasive signal for precision livestock farming because they can reflect animal behaviour, health condition, stress response, and welfare-related states without requiring direct physical contact with the animals [1,2,3,4,5]. Automatic pig-sound recognition can therefore support early warning of abnormal conditions, continuous welfare assessment, and intelligent farm management. Traditional pig vocalization recognition methods usually rely on handcrafted acoustic descriptors, such as Mel-frequency cepstral coefficients (MFCCs), spectral centroid, formant-related features, short-time energy, and other manually designed statistics, followed by shallow classifiers such as support vector machines, hidden Markov models, or back-propagation neural networks [6,7,8]. Although these methods are lightweight and interpretable, their performance depends strongly on feature engineering and preprocessing. In real pig houses, background noise, reverberation, microphone placement, individual variation, and the acoustic similarity among different vocal categories may reduce the robustness of handcrafted features. To overcome these limitations, recent studies have increasingly adopted deep-learning-based methods, which can learn discriminative time–frequency representations directly from waveforms or spectrograms and reduce the dependence on manually designed acoustic features.

Deep-learning-based pig vocalization recognition has achieved promising performance in several representative tasks. Briefer et al. used a spectrogram-based neural network to classify pig calls according to emotional valence and context of production, achieving 91.5% accuracy for valence classification and 81.5% accuracy for context classification [9]. Liao et al. proposed a TransformerCNN model for domestic pig sound classification and reported 96.05% accuracy, 98.37% AUC, and 90.52% recall, showing the advantage of combining convolutional feature extraction with attention-based modelling [10]. Pan et al. introduced a DNN-HMM framework for pig sound recognition and achieved 83% recognition accuracy on a pig-sound dataset, outperforming conventional HMM and GMM-HMM baselines [11]. Pann et al. further developed a DCNN-based pig vocalization and non-vocalization classification method using real pig-farm recordings, where the proposed Mixed-MMCT feature representation achieved 99.50%, 99.56%, and 99.67% accuracy on three farm datasets, and 95.67% average accuracy in cross-farm robustness experiments [12]. These studies demonstrate the effectiveness of neural and deep models for pig-sound analysis. However, most existing results are obtained under relatively sufficient labelled-data settings or fixed training protocols. In practical farm applications, some vocal categories associated with specific behaviours or physiological states are difficult to collect repeatedly and label accurately. Therefore, few-shot pig vocalization recognition, where only a small number of labelled samples are available for each class, is more consistent with realistic deployment conditions.

Recent livestock vocalization and bioacoustic studies also show that labelled farm-animal acoustic datasets are often limited or imbalanced. Jung et al. developed a cattle vocal classification model using 897 classification records [13]. Gavojdian et al. reported 1144 cattle vocalization records in BovineTalk for high-/low-frequency call classification and individual cow identification [14]. Kate and Neethirajan introduced a bovine bioacoustic dataset containing 569 curated clips across 48 behavioural classes, where many classes contained fewer than ten samples [15]. In few-shot bioacoustic event detection, only five exemplar vocalizations are commonly provided for the target class [16]. These studies indicate that limited labelled animal-sound data are common in livestock and bioacoustic applications. Therefore, the present study focuses on a controlled limited-label pig vocalization protocol, where K-shot subsets are randomly selected from the available training pool and evaluated on a balanced fixed test set.

Under low-resource acoustic conditions, existing studies have mainly explored three types of strategies that are relevant to few-shot pig vocalization recognition: data augmentation, transfer learning, and metric learning. Data augmentation aims to enlarge the effective training distribution without collecting additional labelled recordings. Common audio augmentation strategies include pitch shifting, time stretching, time shifting, background-noise injection, random gain perturbation, and sample-mixing methods, which have been used to improve robustness in environmental sound classification, domestic audio tagging, and pig vocalization/non-vocalization classification [12,17,18]. Transfer learning provides another solution by reusing representations learned from large-scale audio corpora. For example, pretrained audio neural networks trained on AudioSet have shown strong transferability across audio tagging, acoustic scene classification, music classification, speech emotion recognition, and sound event detection tasks [19]. Efficient Audio Transformer further demonstrated that self-supervised audio pretraining can achieve strong performance on AudioSet, ESC-50, and speech-command classification while reducing pretraining cost [20]. Metric learning attempts to improve class separability when labelled samples are scarce. Prototypical networks learn a metric space in which classification is performed by distances to class prototypes [21], and relation networks learn a deep comparison function for few-shot recognition [22]. In bioacoustic few-shot event detection, prototypical-network-based baselines and segment-level metric-learning systems have been widely explored; for example, segment-level metric learning substantially improved the F-measure over the DCASE few-shot bioacoustic baseline by making better use of positive and negative acoustic segments [23,24]. Nevertheless, each strategy alone still has limitations. Data augmentation can increase local acoustic variation but cannot provide high-level representation priors. Transfer learning can improve initialization but may not fully adapt the embedding space to target pig vocalization categories. Metric learning can enhance class separation, but its effectiveness is still constrained when the few labelled samples do not sufficiently cover intra-class acoustic variation. These observations motivate an integrated framework that jointly improves sample coverage, representation initialization, and embedding discriminability.

To address these limitations, we propose PSA-AP, a pig-sound adaptation pipeline for few-shot pig vocalization recognition. PSA-AP uses log-Mel spectrograms as input and combines three task-adaptive components. SpecAugment-based domain expansion applies label-preserving time and frequency masking to scarce training spectrograms, thereby increasing the perturbation coverage of the observed samples. ImageNet-pretrained ResNet18 knowledge transfer provides a stable initialization for spectrogram representation learning. ArcFace-based feature alignment encourages intra-class compactness and inter-class separation in the embedding space. Together, these components are intended to reduce representation-learning difficulty, mitigate overfitting, and clarify class boundaries under few-shot supervision.

In this paper, we focus on the few-shot pig vocalization recognition problem and propose PSA-AP as a task-adapted supervised framework for log-Mel spectrogram classification. Specifically, PSA-AP integrates SpecAugment-based domain expansion, ImageNet-pretrained ResNet18 knowledge transfer, and ArcFace-based feature alignment to improve sample coverage, representation initialization, and embedding separability under limited labelled data. The main contributions of this study are summarized as follows:

1. We formulate and evaluate a controlled five-class few-shot pig vocalization recognition protocol covering eat, estrous, farrowing (fap), howl, and oink, with K={5,10,15,20,25,30} labelled wav files per class, five random seeds, and a fixed wav-file-level test set.
2. We propose PSA-AP, a pig-sound adaptation pipeline that combines spectrogram-domain expansion, transferred ResNet18 representation learning, and angular-margin feature alignment to mitigate limited-sample representation learning and unclear class boundaries.
3. We compare PSA-AP with representative model families using the last checkpoint as the final evaluation model and show that, within the current protocol, PSA-AP obtains the highest mean Accuracy, Macro-F1, and UAR across all six K-shot settings, reaching 90.60% Accuracy at K=30.

## 2. Materials and Methods

### 2.1. Dataset Acquisition and Preparation

**Figure 1:**
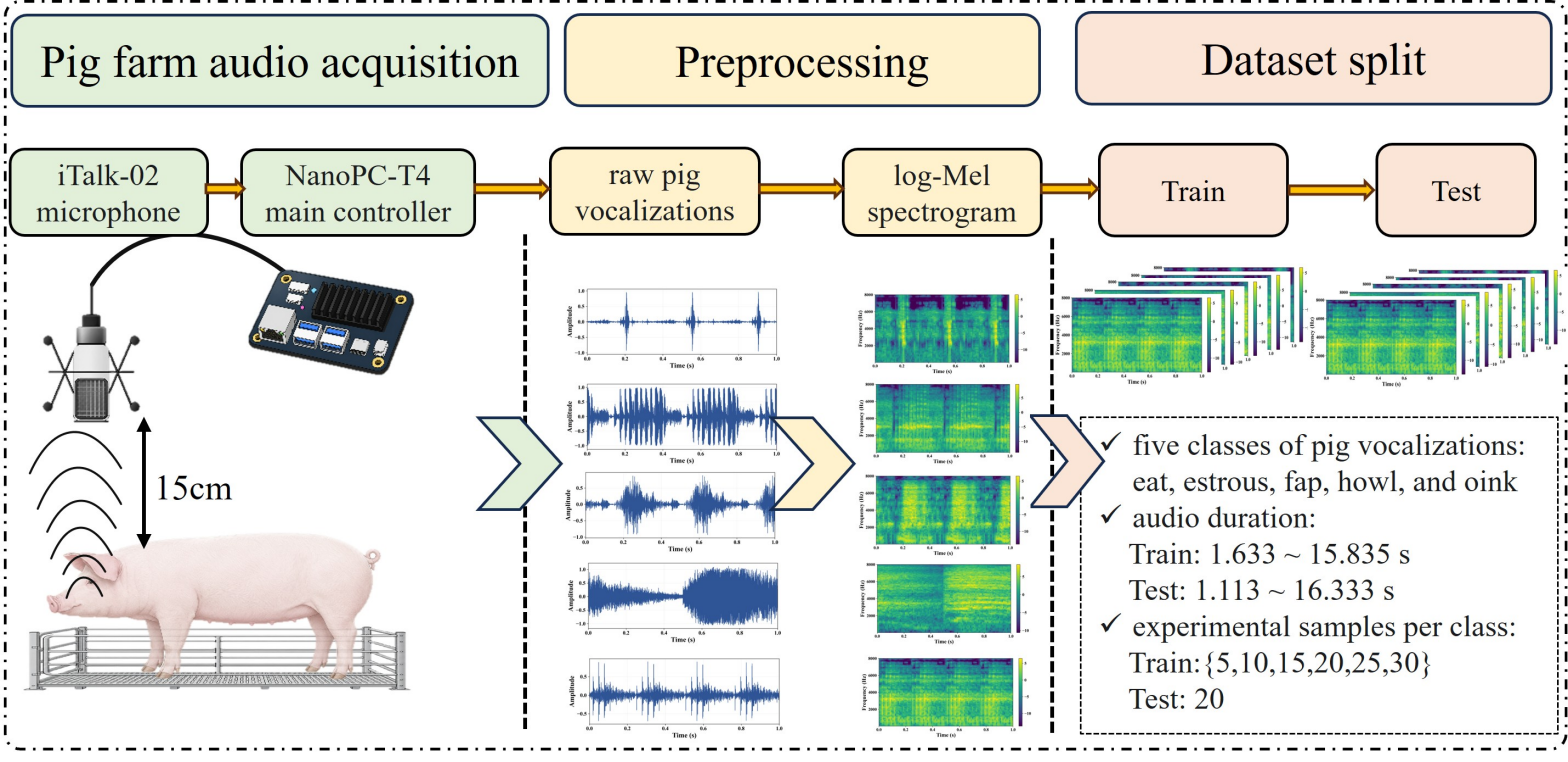
Dataset acquisition and preparation workflow.

Figure 1 summarizes the dataset acquisition and preparation workflow. Audio recordings were collected at Jinghuimeng Pig Farm, Mengcheng, Anhui Province, China. Ten adult Landrace pigs were housed in individual pens placed at relatively large intervals in a quiet sampling space. A microphone was fixed approximately 15 cm to the right side of each pig’s individual pen to collect vocalization signals while reducing interference from adjacent animals and the surrounding environment. Data were collected for five days from 21 February 2024 to 25 February 2024. On each day, recordings were acquired during three time periods: 05:00–08:00, 11:00–14:00, and 17:00–20:00. The recordings were classified and annotated by two researchers and then reviewed by two pig-farm staff members. Recordings with unclear labels, severe background interference, or multiple overlapping vocalization categories were excluded from the retained dataset. In total, 310 wav files with durations ranging from 1.113 to 16.333 s were retained, and each file contained only one sound category.

The audio acquisition system was self-developed for this study. A NanoPC-T4 single-board computer (FriendlyElec, Guangzhou, China) was used as the main controller and was connected to an iTalk-02 microphone (Newline, Beijing, China) and USB interface resources (Ugreen Group Limited, Beijing, China) resources for audio capture and transmission.

**Figure 2:**
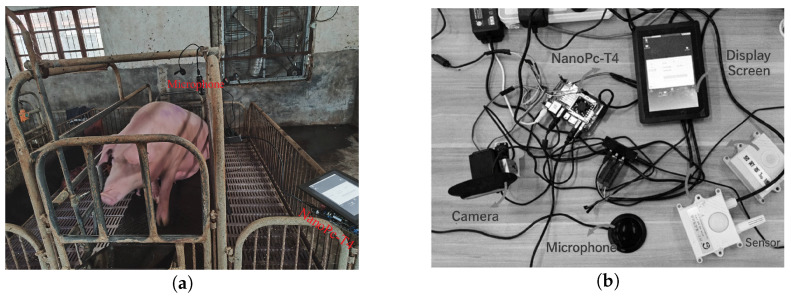
On-site equipment installation and sound acquisition hardware system. Panel (**a**) shows the on-site equipment installation, and panel (**b**) shows the sound acquisition and transmission hardware system.

To obtain single-channel audio signals, the microphone used PCM encoding and exported wav-format recordings with one channel, an 8-bit sample size, and a sampling rate of 44.1 kHz. The on-site equipment installation and the sound acquisition and transmission hardware system are shown in Figure 2.

During preprocessing, each retained wav recording was resampled to 16 kHz and cropped or padded to 1.0 s before feature extraction. Each wav file therefore generated one 1.0 s log-Mel spectrogram for model training or testing, rather than multiple training or test samples. The resulting neural-network input had a shape of 1×128×101, corresponding to one channel, 128 Mel-frequency bins, and 101 time frames. Normalization statistics were fitted separately for each K-shot training subset to avoid using information from the full training pool or the fixed test set.

**Figure 3:**
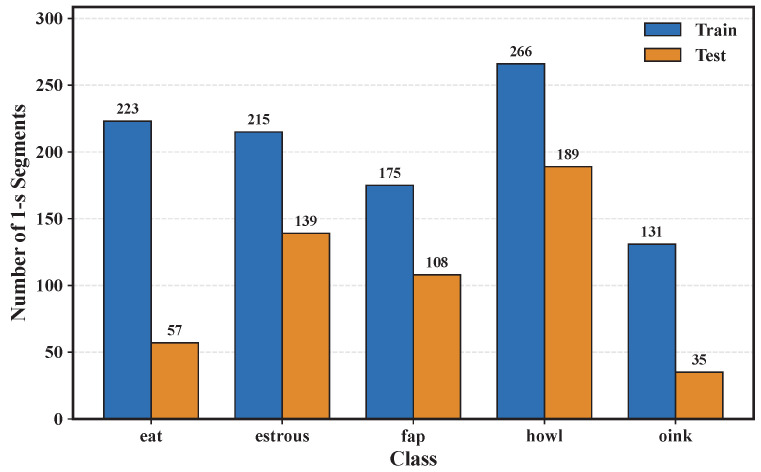
Class-wise complete non-overlapping 1.0 s segment counts used only for describing the available duration distribution of the retained recordings.

The dataset contains five pig vocalization classes: eat, estrous, farrowing (fap), howl, and oink. The training pool contains 42 wav files per class, giving 210 training-pool recordings in total, with durations ranging from 1.633 to 15.835 s. The fixed test set contains 100 wav files, with 20 recordings per class, and durations ranging from 1.113 to 16.333 s. For each few-shot experiment, K-shot subsets were sampled at the wav-file level from the training pool with K∈{5,10,15,20,25,30} labelled wav files per class, and each selected wav file was converted into one 1.0 s log-Mel spectrogram for training. Each fixed test wav file was also converted into one deterministic 1.0 s log-Mel spectrogram for evaluation. In addition, the retained training-pool and test wav files were partitioned into complete non-overlapping 1.0 s slices only for segment-count analysis, yielding 1010 training-pool segments and 528 test-set segments. The segment counts were reported only to describe the available duration distribution of the retained recordings, whereas the few-shot model evaluation was conducted at the wav-file level. The class-wise segment counts are shown in Figure 3. Test labels were parsed from filename keywords.

### 2.2. Problem Description

#### 2.2.1. System Model

The study considers a few-shot pig vocalization recognition system in which short raw audio recordings are converted into time–frequency representations and then classified into behavioural or physiological vocalization categories. Let

(1) C={eat,estrous,fap,howl,oink}

denote the label set, where the number of classes is C=|C|=5 and farrowing (fap) is represented by the short label fap. A labelled sample is represented as $\left(a_{i},y_{i}\right)$, where $a_{i}$ denotes one pig vocalization wav recording and $y_{i}\in C$ is its class label. Each wav recording is resampled, cropped or padded to 1.0 s, converted to one log-Mel spectrogram, and normalized before being passed to the classifier. This preprocessing pipeline is written as

(2) $$x_{i}=\phi \left(a_{i}\right)\in R^{1\times F\times T},$$

where ϕ(·) denotes the resampling, 1.0 s crop or padding, log-Mel feature extraction, and normalization procedure applied to each wav recording, F=128 is the number of Mel-frequency bins, and T=101 is the number of time frames. The supervised neural classifier is defined as

(3) $$f_{\theta }\left(x_{i}\right)=\left(z_{i},h_{i}\right),$$

where θ denotes the trainable model parameters, $z_{i}\in R^{C}$ denotes the classification logits, and $h_{i}\in R^{d}$ denotes the learned embedding feature before the final classification layer. The predicted probability for class *c* is

(4) $$p_{\theta }\left(y=c|x_{i}\right)=\mathrm{softmax}{\left(z_{i}\right)}_{c}=\frac{\exp(z_{i,c})}{\sum_{j=1}^{C}\exp\left(z_{i,j}\right)}.$$

Here, $z_{i,c}$ is the logit assigned to class *c*, and the denominator normalizes the logits across all five pig vocalization classes.

#### 2.2.2. Few-Shot Pig Vocalization Recognition Problem

For a given shot number K∈{5,10,15,20,25,30}, *K* denotes the number of labelled wav files sampled per class from the training pool. Each selected wav file contributes one 1.0 s log-Mel spectrogram, so the available training set is

(5) $$\begin{array}{c} D_{\mathrm{tr}}^{K}={\left\{\left(x_{i},y_{i}\right)\right\}}_{i=1}^{CK}, \end{array}$$

where each class contributes exactly *K* labelled wav-file samples and CK is the total number of training samples. The fixed test set is

(6) $$D_{\mathrm{te}}={\left\{\left(x_{j},y_{j}\right)\right\}}_{j=1}^{N_{\mathrm{te}}},$$

where $N_{\mathrm{te}}=100$ and each element denotes one held-out wav file represented by one deterministic 1.0 s log-Mel spectrogram. The learning objective for a model trained on a K-shot subset can be expressed as empirical risk minimization:

(7) $$\theta^{*}=\arg\min_{\theta }\frac{1}{|D_{\mathrm{tr}}^{K}|}\sum_{\left(x_{i},y_{i}\right)\in D_{\mathrm{tr}}^{K}}L\left(f_{\theta }\left(x_{i}\right),y_{i}\right),$$

where L(·) denotes the training loss and $\theta^{*}$ denotes the fitted model parameters. Because CK is small in this setting, especially at K=5 and K=10, directly learning a high-capacity deep representation from scratch is prone to overfitting and unstable class boundaries. In this study, K=5 and K=10 are regarded as extremely low-label settings, whereas K=15, K=20, K=25, and K=30 are treated as limited-label supervised adaptation settings rather than extreme few-shot settings. The proposed task-adapted supervised method therefore combines domain expansion, knowledge transfer, and metric-learning feature alignment.

### 2.3. Motivation

The methodological design follows a standard empirical-risk-minimization intuition rather than a new theoretical guarantee [25]. With probability at least 1−δ, the gap between the expected risk and the empirical K-shot training risk can be informally motivated as

(8) $$\left|R\left(f_{\theta }\right)-R_{D_{\mathrm{tr}}^{K}}\left(f_{\theta }\right)\right|\leq O\left(\sqrt{\begin{array}{c} \frac{C(H)-\log\delta }{CK} \end{array}}\right),$$

where $R(f_{\theta })$ is the expected risk on the underlying pig vocalization distribution, $R_{D_{\mathrm{tr}}^{K}}\left(f_{\theta }\right)$ is the empirical risk on the selected K-shot wav-file-level training subset, H is the model hypothesis space, C(H) denotes an effective capacity or complexity term, CK is the total number of labelled training wav files, and 1−δ is the confidence level. In this motivating bound, the small denominator CK captures the central difficulty of few-shot pig vocalization recognition: the training set provides limited coverage of within-class acoustic variation and between-class similarity.

This intuition leads to three complementary design choices. First, SpecAugment performs domain expansion by applying time and frequency masking to log-Mel spectrograms during training, thereby exposing the model to local spectral perturbations without changing class labels. Second, ImageNet-pretrained ResNet18 fine-tuning provides knowledge transfer through a transferable initialization, reducing the need to learn all filters from the small pig vocalization set alone. Third, ArcFace angular-margin loss performs metric-learning feature alignment so that samples from the same vocalization class are encouraged to be compact while different classes are separated by a larger angular margin.

### 2.4. Method Overview

**Figure 4:**
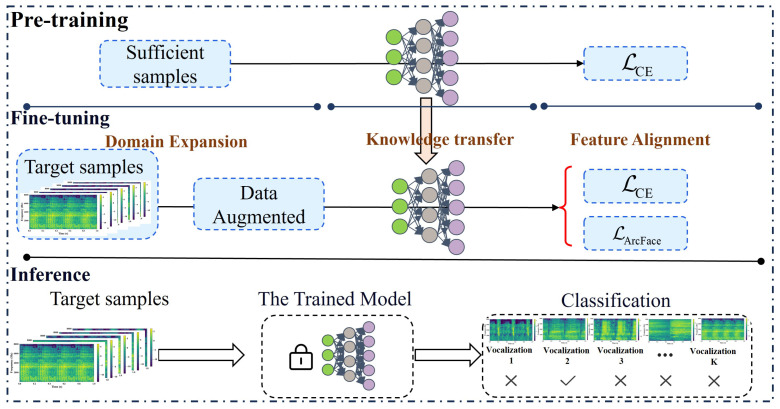
Overview of the proposed few-shot pig vocalization classification framework.

Figure 4 gives an overview of the proposed framework for few-shot pig vocalization classification. Compared with conventional fine-tuning under sufficient labelled samples, the proposed pipeline addresses limited target samples through three task-adaptive components: domain expansion, knowledge transfer, and metric-learning feature alignment. During training, target log-Mel spectrograms are augmented, passed through a transferred ResNet18 backbone, and optimized using cross-entropy together with ArcFace supervision. During inference, the trained model directly classifies fixed test spectrograms into the five pig vocalization categories. The adapted ResNet18 structure used in this study is summarized in Table 1.

### 2.5. SpecAugment Domain Expansion

**Figure 5:**
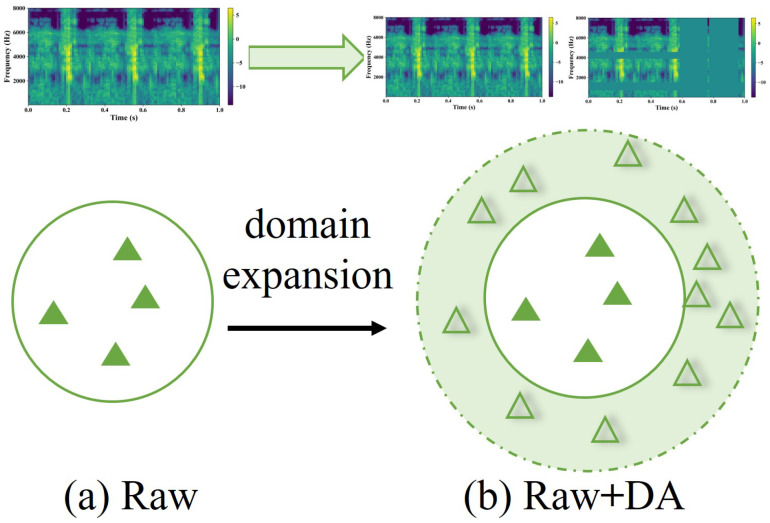
SpecAugment-based domain expansion for few-shot pig vocalization classification.

The first task-adaptive component is SpecAugment-based domain expansion, illustrated in Figure 5. In few-shot pig vocalization recognition, each class provides only a small number of target spectrograms, so the observed training samples cover only a limited region of the possible acoustic variation. SpecAugment expands this target domain by generating label-preserving time–frequency perturbations of the available log-Mel spectrograms during training, while the evaluation pipeline remains deterministic and unaugmented.

For a log-Mel spectrogram $X_{i}=\phi \left(a_{i}\right)\in R^{F\times T}$ with label $y_{i}$, the augmented sample is defined as

(9) $${\tilde{X}}_{i}=A_{\mathrm{SA}}\left(X_{i};M_{f},M_{t}\right),\;{\tilde{y}}_{i}=y_{i},$$

where $A_{\mathrm{SA}}\left(\cdot \right)$ denotes the SpecAugment operator, and $M_{f}$ and $M_{t}$ denote the frequency- and time-masking operations. Specifically, for randomly selected starting positions $f_{0}$ and $t_{0}$ and mask widths $f_{m}$ and $t_{m}$, the masking process is implemented as

(10) $${\tilde{X}}_{i,f_{0}:f_{0}+f_{m},:}=v,\;{\tilde{X}}_{i,:,t_{0}:t_{0}+t_{m}}=v,$$

where *v* is the mask value in the pre-normalized log-Mel space. In this study, *v* was set to the mean value of the corresponding spectrogram, producing a neutral masked region without introducing a fixed out-of-distribution constant. The augmented training set can therefore be viewed as

(11) $${\tilde{D}}_{\mathrm{tr}}^{K}=D_{\mathrm{tr}}^{K}\cup \left\{\left({\tilde{X}}_{i},y_{i}\right)|\left(X_{i},y_{i}\right)\in D_{\mathrm{tr}}^{K}\right\},$$

which increases the effective coverage of local temporal and spectral variations around the observed pig vocalization samples. This domain expansion is applied only during training and does not change the class labels.

### 2.6. Knowledge Transfer

**Figure 6:**
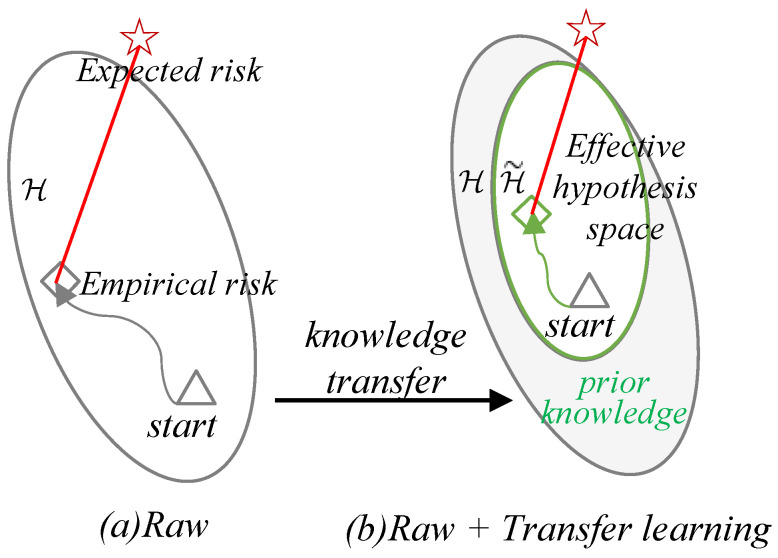
Knowledge transfer through ImageNet-pretrained ResNet18 fine-tuning.

The second task-adaptive component is knowledge transfer through ImageNet-pretrained ResNet18 fine-tuning, as illustrated in Figure 6. In the raw few-shot setting, model optimization starts from random parameters and searches a large hypothesis space using only a small number of pig vocalization samples. By contrast, transfer learning introduces prior knowledge through pretrained convolutional filters and initializes the model in a more effective region of the hypothesis space, reducing the burden of learning all spectrogram representations from the K-shot target set alone.

Let $\theta_{\mathrm{pre}}$ denote the parameters of a ResNet18 model pretrained on ImageNet [26]. The pig vocalization classifier is initialized from these parameters and then adapted on the K-shot training set:

(12) $$\theta^{*}=\arg\min_{\theta }\frac{1}{|D_{\mathrm{tr}}^{K}|}\sum_{\left(x_{i},y_{i}\right)\in D_{\mathrm{tr}}^{K}}L_{\mathrm{KT}}\left(f_{\theta }\left(x_{i}\right),y_{i}\right),\;\theta_{0}\leftarrow \theta_{\mathrm{pre}},$$

where $\theta_{0}$ is the initialization used before fine-tuning, and $L_{\mathrm{KT}}$ denotes the supervised fine-tuning loss. Although ImageNet consists of natural images rather than animal sounds, its early convolutional filters provide transferable edge- and texture-like primitives that are also useful for spectrogram-like inputs. This transfer is especially important when the target pig vocalization set is too small to learn robust low-level filters from scratch.

Because the pretrained ResNet18 expects three RGB input channels whereas the log-Mel spectrogram has one channel, the first convolutional layer was adapted by averaging the three input-channel kernels:

(13) $${\tilde{W}}_{o,1,:,:}^{\left(1\right)}=\frac{1}{3}\sum_{r=1}^{3}W_{o,r,:,:}^{\left(1\right)},\;o=1,\ldots ,64,$$

where $W^{\left(1\right)}$ denotes the original first-layer convolutional weight tensor, ${\tilde{W}}^{\left(1\right)}$ denotes the adapted one-channel weight tensor, *o* indexes the output convolutional channel, and *r* indexes the original RGB input channels. The final classification layer was replaced with a five-class output layer. For each input $x_{i}$, the network returns logits $z_{i}$ for classification and an embedding $h_{i}$ for visualization and metric-learning supervision. ResNet18 trained from scratch was included only as an ablation control to quantify the value of pretraining; it is not the main proposed method.

### 2.7. Metric-Learning Feature Alignment

**Figure 7:**
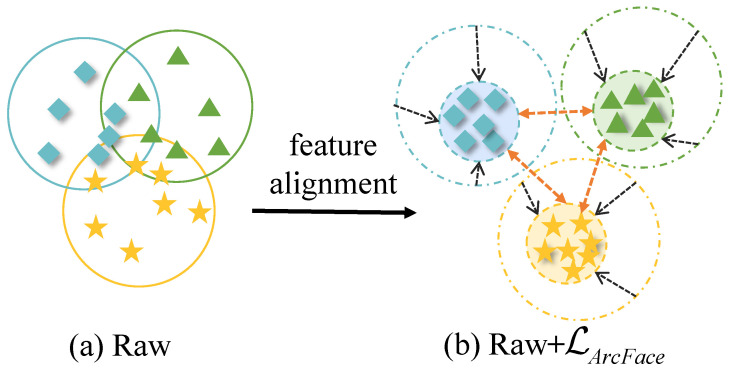
Metric-learning feature alignment using ArcFace angular-margin supervision.

The third task-adaptive component is metric-learning feature alignment using ArcFace angular-margin supervision, as shown in Figure 7. In few-shot pig vocalization classification, acoustically related categories may produce overlapping embeddings when the model is trained only with a standard classification objective. ArcFace reduces this overlap by pulling embeddings of the same vocalization class toward a compact angular region and pushing different classes apart in the normalized feature space.

For an input spectrogram $x_{i}$, the backbone produces an embedding $h_{i}\in R^{d}$. ArcFace first normalizes both the embedding and class weight vectors:

(14) $${\bar{h}}_{i}=\frac{h_{i}}{∥h_{i}{∥}_{2}},\;{\bar{w}}_{j}=\frac{w_{j}}{∥w_{j}{∥}_{2}},\;\cos\theta_{j}={\bar{w}}_{j}^{⊤}{\bar{h}}_{i},$$

where ${\bar{h}}_{i}$ is the normalized feature, ${\bar{w}}_{j}$ is the normalized prototype weight for class *j*, and $\theta_{j}$ is the angle between sample *i* and class *j*. For the ground-truth class $y_{i}$, an additive angular margin *m* is imposed before the softmax operation:

(15) $$L_{\mathrm{ArcFace}}=-\frac{1}{B}\sum_{i=1}^{B}\log\frac{\exp\left(s\cos(\theta_{y_{i}}+m)\right)}{\exp\left(s\cos(\theta_{y_{i}}+m)\right)+\sum_{j\neq y_{i}}\exp\left(s\cos\theta_{j}\right)}.$$

Here, *B* denotes the batch size and *s* is a scale factor that stabilizes optimization after feature normalization. The margin *m* makes the correct-class decision stricter in angular space, encouraging more compact within-class clusters and clearer separation between pig vocalization classes.

The final training objective combines conventional class-label supervision with metric-learning feature alignment:

(16) $$L=L_{\mathrm{CE}}+\lambda_{\mathrm{arc}}L_{\mathrm{ArcFace}},$$

where $L_{\mathrm{CE}}$ is the cross-entropy loss computed from the classification logits, and $\lambda_{\mathrm{arc}}$ controls the contribution of the ArcFace branch. Cross-entropy preserves stable supervised classification, whereas ArcFace aligns the embedding space so that few-shot pig vocalization features become more class compact and less mutually confused.

## 3. Results

### 3.1. Experimental Settings

All experiments followed the same few-shot evaluation protocol. For each method, the training subset was sampled at the wav-file level from the training pool using K∈{5,10,15,20,25,30} labelled wav files per class and five random seeds. Each selected wav file was converted into one 1.0 s log-Mel spectrogram for model training. The fixed test set contained 100 held-out wav files, with 20 wav files per class, and each test wav file was converted into one deterministic 1.0 s log-Mel spectrogram for model evaluation. For neural-network methods, training was run for 100 epochs without early stopping. The last checkpoint from the final epoch was used as the final evaluation model in all reported results. This policy avoids selecting models by the fixed-test trajectory and reflects the performance of the completed training schedule. The main experimental settings are summarized in Table 2.

Let $N_{\mathrm{te}}$ denote the number of test samples, C=5 the number of classes, $y_{i}$ the true label of sample *i*, and ${\hat{y}}_{i}$ the predicted label. The overall accuracy is defined as

(17) $$\mathrm{Accuracy}=\begin{array}{c} \frac{1}{N_{\mathrm{te}}}\sum_{i=1}^{N_{\mathrm{te}}}I\left({\hat{y}}_{i}=y_{i}\right), \end{array}$$

where I(·) is the indicator function. For class *c*, the per-class accuracy is

(18) $${\mathrm{Acc}}_{c}=\frac{1}{N_{c}}\sum_{i:y_{i}=c}I\left({\hat{y}}_{i}=y_{i}\right),$$

where $N_{c}$ is the number of test samples belonging to class *c*. Precision, recall, and F1-score for class *c* are computed as

(19) $$P_{c}=\frac{{\mathrm{TP}}_{c}}{{\mathrm{TP}}_{c}+{\mathrm{FP}}_{c}},\;R_{c}=\frac{{\mathrm{TP}}_{c}}{{\mathrm{TP}}_{c}+{\mathrm{FN}}_{c}},\;\begin{array}{c} F1_{c}=\frac{2P_{c}R_{c}}{P_{c}+R_{c}}, \end{array}$$

where ${\mathrm{TP}}_{c}$, ${\mathrm{FP}}_{c}$, and ${\mathrm{FN}}_{c}$ denote true positives, false positives, and false negatives for class *c*, respectively. Macro-F1 and unweighted average recall (UAR) are then given by

(20) $$\mathrm{Macro}-F1=\begin{array}{c} \frac{1}{C}\sum_{c=1}^{C}F1_{c}, \end{array}\;\mathrm{UAR}=\frac{1}{C}\sum_{c=1}^{C}R_{c}.$$

Because the fixed wav-file-level test set is class-balanced with 20 wav files per class, Accuracy and UAR can have the same numerical value in this study. Nevertheless, UAR is retained to explicitly report class-balanced recall and to facilitate comparison with studies using imbalanced test sets.

### 3.2. Comparison Models

Five methods were compared in the main experiment, and only their abbreviations are used hereafter. Raw denotes the baseline ResNet18 architecture trained from random initialization on the K-shot log-Mel spectrograms [26]. MFCC-SVM denotes a traditional acoustic machine-learning baseline that summarizes each recording using mel-frequency cepstral coefficients [6] and trains a support vector machine classifier [7]. Small-CNN denotes a compact convolutional neural network trained from scratch on log-Mel spectrograms, following the common CNN-based environmental-sound classification paradigm [27]. EAT-FL denotes an external frozen Efficient Audio Transformer representation with only a linear classifier fitted to the K-shot pig vocalization subset [20]. PSA-AP denotes the proposed pig-sound adaptation pipeline, which combines pig vocalization spectrogram augmentation, ImageNet-pretrained ResNet18 knowledge transfer, and ArcFace angular-margin prototype/feature alignment [26,28,29].

### 3.3. Recognition Performance of Different Models

**Figure 8:**
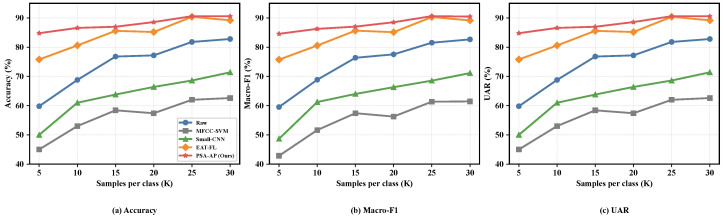
Recognition performance across K-shot settings using the last checkpoint. Subfigures show (**a**) Accuracy, (**b**) Macro-F1, and (**c**) UAR for the five comparison methods. Each point represents the mean result over five random seeds at the corresponding value of *K*.

Figure 8 compares the Accuracy, Macro-F1, and UAR curves of the five recognition methods as the number of labelled wav files per class increased from K=5 to K=30, and the numerical values corresponding to the figure are formally reported in Table 3. All values are based on the last checkpoint of each training run and were computed on the fixed 100-wav-file test set. MFCC-SVM consistently produced the lowest performance, with accuracy increasing from 45.00% to 62.60%. Small-CNN improved over MFCC-SVM at every K-shot setting and reached 71.40% accuracy at K=30, indicating that log-Mel spectrogram learning was more effective than handcrafted cepstral summaries. Raw further improved the results, rising from 59.80% to 82.80%, which shows that the residual architecture extracted more discriminative time-frequency features even without external pretraining.

PSA-AP achieved the highest mean performance across all six K-shot settings for the three metrics. At K=5, PSA-AP reached 84.80% accuracy, 84.58% Macro-F1, and 84.80% UAR, exceeding EAT-FL by 9.00, 8.83, and 9.00 percentage points, respectively. At K=30, PSA-AP reached 90.60% accuracy, 90.49% Macro-F1, and 90.60% UAR, exceeding Raw by 7.80, 7.82, and 7.80 percentage points, respectively. The consistent advantage across Accuracy, Macro-F1, and UAR indicates that the proposed combination of pig vocalization spectrogram augmentation, knowledge transfer, and angular-margin feature alignment improved both overall correctness and class-balanced recognition performance. These results support the feasibility of PSA-AP as a task-adapted supervised framework for few-shot pig vocalization recognition.

### 3.4. Discrimination Analysis of Feature Extraction Ability

**Figure 9:**
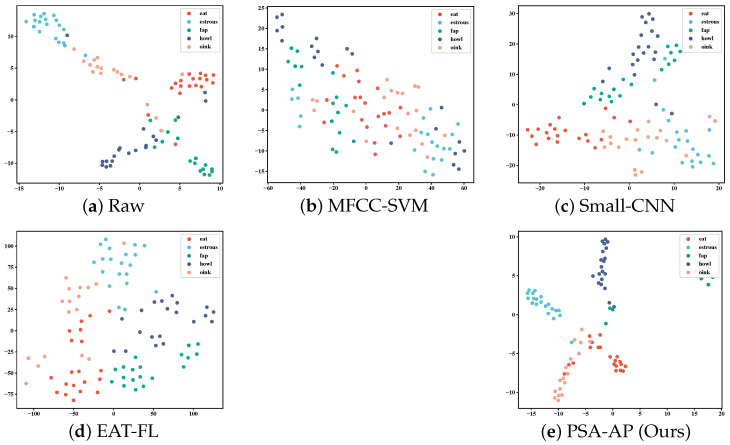
t-SNE visualization of feature extraction ability for different recognition models at K=30 and seed 42 using last-checkpoint features. Panels are ordered as Raw, MFCC-SVM, Small-CNN, EAT-FL, and PSA-AP (Ours). Each point denotes one fixed test-set wav-file sample and colours indicate the true vocalization class.

To further examine the feature extraction ability of different recognition models, t-SNE visualizations were generated from the K=30, seed 42 test-set features using the last checkpoint. As shown in Figure 9, the panels are ordered as Raw, MFCC-SVM, Small-CNN, EAT-FL, and PSA-AP (Ours). Raw and MFCC-SVM produced mixed distributions with limited class-discriminative structure. Small-CNN showed partial separation, but several categories still overlapped in the projected space. EAT-FL formed a clearer global structure than the traditional and small-network baselines, which is consistent with the benefit of transferable audio representations under limited labels.

PSA-AP generated the most compact and clearly separated clusters among the five methods. This pattern indicates that pig vocalization spectrogram augmentation, ImageNet-based knowledge transfer, and ArcFace angular-margin alignment jointly enhanced the discriminative structure of the embedding space. The visual separation in the last-checkpoint feature space provides direct evidence that the proposed method is feasible for learning task-adapted features, rather than merely improving the scalar performance metrics.

### 3.5. Stability Analysis

**Figure 10:**
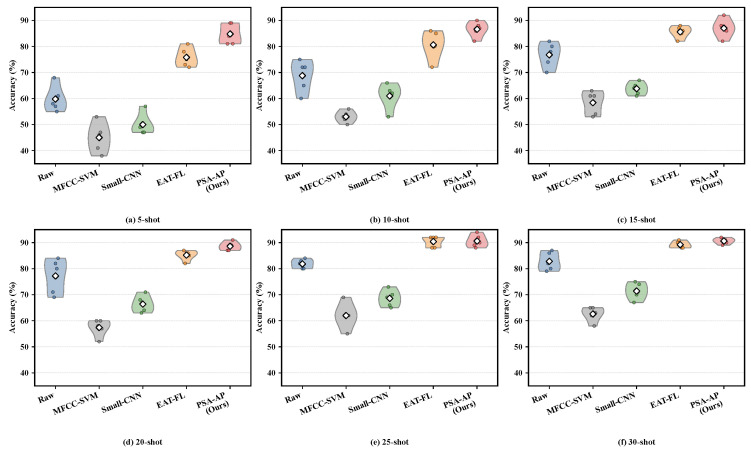
Accuracy stability analysis under all six K-shot settings using the last checkpoint. Violin plots show the distribution across five random seeds for each recognition method; white diamonds denote mean accuracy. The upper row contains 5-shot, 10-shot, and 15-shot settings, and the lower row contains 20-shot, 25-shot, and 30-shot settings.

Stability across random few-shot sampling is important for evaluating whether a recognition method can be reliably used when only a small number of labelled pig vocalization samples are available. Therefore, the accuracy distributions over five random seeds were visualized for all six K-shot settings. As shown in Figure 10, the 5-shot, 10-shot, and 15-shot settings are placed in the upper row, and the 20-shot, 25-shot, and 30-shot settings are placed in the lower row. MFCC-SVM and Small-CNN remained at relatively low accuracy levels, and Raw showed seed-dependent variation despite its higher mean accuracy, indicating that directly training supervised models from limited pig vocalization samples can lead to unstable performance when the selected K-shot subset changes.

The proposed PSA-AP method showed a more desirable stability pattern across the full label range. PSA-AP obtained the highest mean accuracy in every K-shot setting and became more concentrated as the number of labelled samples increased. In the 30-shot setting, PSA-AP achieved 90.60 ± 1.14% accuracy, while EAT-FL reached 89.20 ± 1.30%. This compact distribution, together with the highest mean accuracy, indicates that PSA-AP not only improves recognition performance but also reduces sensitivity to few-shot sampling variation. The stability advantage further supports the feasibility of combining pig vocalization spectrogram augmentation, knowledge transfer, and angular-margin feature alignment for robust few-shot pig vocalization recognition.

### 3.6. Ablation Experiment

The ablation experiment was used to examine the contribution of the three components in PSA-AP: knowledge transfer (KT), SpecAugment-based domain expansion (DE), and ArcFace-based feature alignment (FA). Raw denotes ResNet18 trained from scratch without these components. KT denotes ImageNet-pretrained ResNet18 fine-tuning. KT+DE and KT+FA denote adding SpecAugment or ArcFace to the KT backbone, respectively. PSA-AP combines all three components. Table 4 reports Accuracy, Macro-F1, and UAR for all six K-shot settings using the last checkpoint. Across the full range from 5 to shot to 30-shot, KT improved substantially over Raw, and adding DE or FA generally provided further gains over KT. The full PSA-AP achieved the best value for every metric and every K-shot setting, reaching 90.60% Accuracy, 90.49% Macro-F1, and 90.60% UAR at K=30.

**Figure 11:**
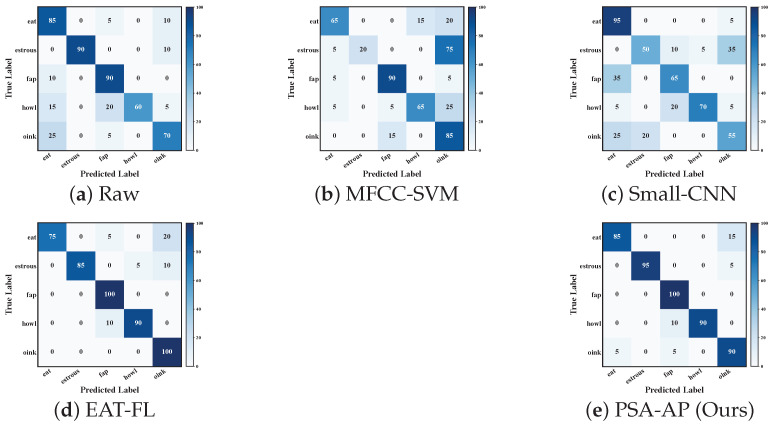
Row-normalized confusion matrices of the five recognition models at K=30 and seed 42 using the last checkpoint. Panels are ordered as Raw, MFCC-SVM, Small-CNN, EAT-FL, and PSA-AP (Ours). Values indicate class-wise recognition accuracy in percentages.

The row-normalized confusion matrices in Figure 11 further compare the class-wise behaviour of the five recognition models under the 30-shot, seed 42 setting using the last checkpoint. Raw, MFCC-SVM, and Small-CNN showed more off-diagonal errors, indicating that conventional handcrafted features or purely scratch-trained compact networks had difficulty separating all five pig vocalization categories. EAT-FL improved the diagonal structure through external audio representations, but some class confusion remained. PSA-AP preserved the strongest diagonal-dominant pattern among the five models, supporting the feasibility of integrating spectrogram-domain expansion, transferred representation learning, and angular-margin feature alignment for discriminative few-shot pig vocalization recognition.

## 4. Discussion

This study shows that the proposed PSA-AP framework is feasible for few-shot pig vocalization classification when the last checkpoint is used as the final evaluation model. As shown in Figure 8 and Table 3, PSA-AP achieved the highest mean Accuracy, Macro-F1, and UAR across all six K-shot settings. The advantage was already evident at 5-shot and remained stable through 30-shot, where PSA-AP reached 90.60% Accuracy, 90.49% Macro-F1, and 90.60% UAR. The central finding is therefore that combining domain expansion, knowledge transfer, and feature alignment can produce reliable task-adapted supervised learning even under limited labelled pig-sound data [30,31,32,33,34,35,36,37,38,39,40,41].

The comparison among MFCC-SVM, Small-CNN, Raw, EAT-FL, and PSA-AP further clarifies why task-adapted representation learning is important for few-shot pig vocalization recognition. MFCC-SVM produced the lowest performance, suggesting that handcrafted cepstral summaries were insufficient to describe the complex time-frequency differences among the five pig vocalization categories. Small-CNN improved over MFCC-SVM, indicating that local structures in log-Mel spectrograms contain useful discriminative information. Raw achieved stronger results than Small-CNN, but its remaining gap to PSA-AP suggests that training a deeper residual network from limited pig-specific samples still faces representation learning and overfitting challenges. EAT-FL demonstrated the benefit of external audio representations, whereas PSA-AP showed that supervised pig-specific adaptation can further organize the representation space when paired with augmentation and angular-margin alignment [42,43].

The expanded ablation results in Table 4 support the complementary roles of the three PSA-AP components across all label budgets. ImageNet-based knowledge transfer provides ResNet18 with a more stable low-level initialization, reducing the burden of learning all filters from a small pig vocalization subset. SpecAugment-based domain expansion introduces time and frequency masking in the log-Mel input space, which may reduce dependence on narrow local spectral regions and improve robustness to temporal and spectral variation. ArcFace-based feature alignment encourages more compact within-class embeddings and larger angular separation between classes, helping form clearer decision boundaries under few-shot supervision. The fact that PSA-AP achieved the best values across all K-shot settings indicates that these components contribute through different but compatible mechanisms rather than acting as redundant modifications.

The feature visualization, stability analysis, and confusion matrices provide additional support for this interpretation. In Figure 9, PSA-AP produced more compact and better separated class clusters than the comparison methods at 30-shot seed 42, indicating stronger feature extraction ability for the five pig vocalization categories. In Figure 10, PSA-AP showed the highest mean accuracy across the 5-shot to 30-shot settings and a compact distribution at higher K values, suggesting reduced sensitivity to the random selection of few-shot training samples. In Figure 11, PSA-AP also showed the clearest diagonal-dominant class-wise recognition pattern. Together, these results indicate that PSA-AP is not only numerically stronger but also more discriminative and stable under the current last-checkpoint evaluation protocol.

**Figure 12:**
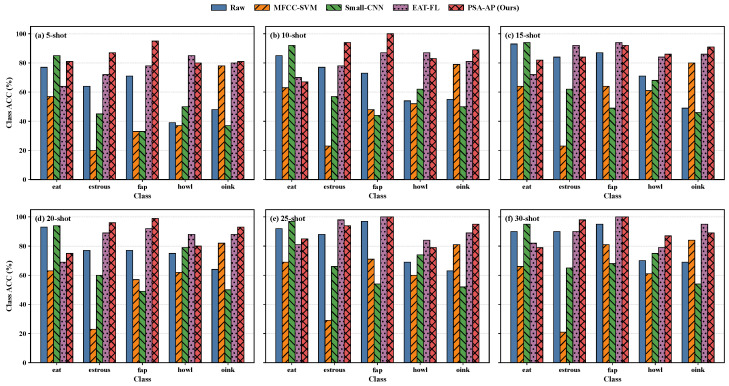
Class-wise average accuracy comparison across the six K-shot settings using the last checkpoint. Panels show (**a**) 5-shot, (**b**) 10-shot, (**c**) 15-shot, (**d**) 20-shot, (**e**) 25-shot, and (**f**) 30-shot results. Bars are ordered as Raw, MFCC-SVM, Small-CNN, EAT-FL, and PSA-AP (Ours) for each pig vocalization class.

The class-wise average accuracy results in Figure 12 further show how the overall performance advantage was distributed among the five pig vocalization categories. PSA-AP maintained high accuracy for estrous and farrowing sounds across most K-shot settings and reached 98.0% and 100.0%, respectively, at 30-shot. It also remained competitive for howl and oink, although EAT-FL or Small-CNN occasionally achieved higher accuracy for individual classes such as eat or oink. This class-level pattern indicates that the improvement of PSA-AP was not caused by a single dominant category alone. Instead, the proposed combination of spectrogram-domain expansion, transferred representation learning, and angular-margin alignment provided a more balanced class-wise recognition profile under limited labelled pig-sound data, which is important for practical monitoring scenarios where rare physiological or behavioural vocalizations should not be missed.

Several limitations should be considered when interpreting these results. The dataset is still small, containing recordings from 10 adult Landrace pigs and five predefined vocalization categories collected from a single farm with a specific recording system. Therefore, generalization across farms, microphones, pig breeds, age groups, and complex noise conditions remains to be verified. In addition, although using the last checkpoint avoids fixed-test checkpoint selection, the current test set is still fixed; future work should introduce an independent validation set and external test set for model selection and generalization assessment. Although the pigs were described as adult Landrace pigs, more detailed demographic information, such as sex, body weight, and recording period, should be reported in future multi-farm studies to support more detailed biological interpretation. Future studies should extend the dataset to more diverse production scenarios and investigate the relationships between vocalization categories, behaviour, physiological state, and animal-welfare indicators [44,45].

## 5. Conclusions

This study proposed PSA-AP for few-shot pig vocalization recognition, integrating SpecAugment-based domain expansion, ImageNet-pretrained ResNet18 knowledge transfer, and ArcFace-based feature alignment on log-Mel spectrograms. Under a fixed five-class protocol with six *K* values and five random seeds, where *K* denotes labelled wav files per class and evaluation uses a fixed 100-wav-file test set, PSA-AP achieved the highest mean Accuracy, Macro-F1, and UAR across all K-shot settings. It reached 90.60% Accuracy, 90.49% Macro-F1, and 90.60% UAR at K=30, and the t-SNE visualization, stability analysis, expanded ablation table, and confusion matrices consistently supported its feasibility for discriminative and stable limited-label pig vocalization recognition. Overall, PSA-AP provides a practical supervised adaptation strategy for few-shot pig vocalization recognition. Future work should validate its generalization using data from multiple farms, recording devices, animals, and independent external test sets.

## Figures and Tables

**Table 1:** Adapted ResNet18 architecture used for log-Mel spectrogram classification. Output sizes are shown for an input spectrogram of 1×128×101.

Stage	Main Operation	Output Size
Input	Log-Mel spectrogram	1×128×101
Conv1	7×7 conv, 64, stride 2	64×64×51
MaxPool	3×3 max pooling, stride 2	64×32×26
Layer1	2 BasicBlocks, 64 channels	64×32×26
Layer2	2 BasicBlocks, 128 channels	128×16×13
Layer3	2 BasicBlocks, 256 channels	256×8×7
Layer4	2 BasicBlocks, 512 channels	512×4×4
AvgPool	Adaptive global average pooling	512×1×1
Embedding	Flatten	512
Classifier	Fully connected layer	5

**Table 2:** Experimental settings used for few-shot pig vocalization classification.

Category	Hyper-Parameter	Value
Dataset	Dataset	Pig vocalization dataset collected at Jinghuimeng Pig Farm
	Number of categories	5
	Classes	eat, estrous, farrowing (fap), howl, and oink
	Input representation	One 1.0 s log-Mel spectrogram per wav file, 1×128×101
	Training pool	42 wav files per class, 210 wav files in total
	Test set	20 wav files per class, 100 wav files in total
	K-shot settings	K∈{5,10,15,20,25,30} labelled wav files per class
Training	Maximum epochs	100
	Early stopping	Disabled
	Checkpoint policy	Last checkpoint from epoch 100 used for final evaluation
	Random seeds	42, 123, 456, 789, 1024
	Batch size	min(5K,32)
	Optimizer	Adam
	Learning rate	$1.0\times 10^{-4}$
	Weight decay	$1.0\times 10^{-4}$
	Scheduler	Cosine annealing, $\eta_{\min}=1.0\times 10^{-6}$
Platform	Python version	3.10.18
	PyTorch version	2.7.1 + CUDA 12.8
	GPU	NVIDIA RTX 5070 Ti Laptop, 12 GB

**Table 3:** Main recognition performance across K-shot settings. Values are percentages and are reported as mean ± standard deviation over five random seeds using the last checkpoint. The best value for each metric and K-shot setting is shown in bold.

Metric	Method	5-Shot	10-Shot	15-Shot	20-Shot	25-Shot	30-Shot
Accuracy	Raw	59.80 ± 5.07	68.80 ± 6.14	76.80 ± 4.82	77.20 ± 6.76	81.80 ± 1.79	82.80 ± 3.56
	MFCC-SVM	45.00 ± 5.79	53.00 ± 2.24	58.40 ± 4.56	57.40 ± 3.29	62.00 ± 4.95	62.60 ± 2.88
	Small-CNN	50.00 ± 4.12	61.00 ± 4.85	63.80 ± 2.39	66.40 ± 3.21	68.60 ± 3.21	71.40 ± 3.21
	EAT-FL	75.80 ± 3.70	80.60 ± 5.55	85.60 ± 2.30	85.20 ± 1.92	90.40 ± 2.19	89.20 ± 1.30
	PSA-AP	**84.80 ± 4.02**	**86.60 ± 2.97**	**87.00 ± 3.61**	**88.60 ± 1.67**	**90.60 ± 2.41**	**90.60 ± 1.14**
Macro-F1	Raw	59.53 ± 4.57	68.86 ± 6.29	76.38 ± 5.15	77.55 ± 6.80	81.53 ± 1.67	82.67 ± 3.41
	MFCC-SVM	42.84 ± 6.56	51.64 ± 2.21	57.41 ± 4.34	56.26 ± 3.39	61.35 ± 5.46	61.45 ± 2.82
	Small-CNN	48.64 ± 3.68	61.23 ± 5.40	64.00 ± 2.53	66.32 ± 3.36	68.54 ± 3.24	71.14 ± 3.26
	EAT-FL	75.75 ± 3.90	80.57 ± 5.25	85.67 ± 2.34	85.14 ± 1.96	90.31 ± 2.28	89.16 ± 1.23
	PSA-AP	**84.58 ± 4.40**	**86.28 ± 3.09**	**87.04 ± 3.55**	**88.54 ± 1.80**	**90.62 ± 2.43**	**90.49 ± 1.21**
UAR	Raw	59.80 ± 5.07	68.80 ± 6.14	76.80 ± 4.82	77.20 ± 6.76	81.80 ± 1.79	82.80 ± 3.56
	MFCC-SVM	45.00 ± 5.79	53.00 ± 2.24	58.40 ± 4.56	57.40 ± 3.29	62.00 ± 4.95	62.60 ± 2.88
	Small-CNN	50.00 ± 4.12	61.00 ± 4.85	63.80 ± 2.39	66.40 ± 3.21	68.60 ± 3.21	71.40 ± 3.21
	EAT-FL	75.80 ± 3.70	80.60 ± 5.55	85.60 ± 2.30	85.20 ± 1.92	90.40 ± 2.19	89.20 ± 1.30
	PSA-AP	**84.80 ± 4.02**	**86.60 ± 2.97**	**87.00 ± 3.61**	**88.60 ± 1.67**	**90.60 ± 2.41**	**90.60 ± 1.14**

**Table 4:** Ablation results across all K-shot settings using the last checkpoint. Values are percentages, mean ± standard deviation over five seeds. KT, DE, and FA denote knowledge transfer, domain expansion, and feature alignment, respectively. Check marks indicate included components, and crosses indicate omitted components. The best value for each metric and K-shot setting is shown in bold.

Metric	Method	KT	DE	FA	5-Shot	10-Shot	15-Shot	20-Shot	25-Shot	30-Shot
Accuracy	Raw	×	×	×	59.80 ± 5.07	68.80 ± 6.14	76.80 ± 4.82	77.20 ± 6.76	81.80 ± 1.79	82.80 ± 3.56
	KT	✓	×	×	71.40 ± 3.71	78.20 ± 3.27	83.60 ± 2.70	80.80 ± 1.30	86.00 ± 3.81	87.00 ± 2.92
	KT+DE	✓	✓	×	74.00 ± 3.54	80.20 ± 3.83	85.80 ± 2.68	85.00 ± 2.35	89.40 ± 3.36	88.00 ± 2.00
	KT+FA	✓	×	✓	73.20 ± 2.77	78.60 ± 4.39	85.80 ± 3.56	82.40 ± 1.14	88.60 ± 3.85	88.60 ± 3.97
	PSA-AP	✓	✓	✓	**84.80 ± 4.02**	**86.60 ± 2.97**	**87.00 ± 3.61**	**88.60 ± 1.67**	**90.60 ± 2.41**	**90.60 ± 1.14**
Macro-F1	Raw	×	×	×	59.53 ± 4.57	68.86 ± 6.29	76.38 ± 5.15	77.55 ± 6.80	81.53 ± 1.67	82.67 ± 3.41
	KT	✓	×	×	71.46 ± 3.52	78.04 ± 3.24	83.59 ± 2.64	80.84 ± 1.26	85.97 ± 3.89	86.98 ± 2.98
	KT+DE	✓	✓	×	73.89 ± 3.61	80.16 ± 3.70	85.81 ± 2.57	84.86 ± 2.47	89.29 ± 3.33	87.96 ± 1.95
	KT+FA	✓	×	✓	73.19 ± 3.34	78.62 ± 4.86	85.66 ± 3.73	82.53 ± 1.21	88.54 ± 3.82	88.63 ± 3.86
	PSA-AP	✓	✓	✓	**84.58 ± 4.40**	**86.28 ± 3.09**	**87.04 ± 3.55**	**88.54 ± 1.80**	**90.62 ± 2.43**	**90.49 ± 1.21**
UAR	Raw	×	×	×	59.80 ± 5.07	68.80 ± 6.14	76.80 ± 4.82	77.20 ± 6.76	81.80 ± 1.79	82.80 ± 3.56
	KT	✓	×	×	71.40 ± 3.71	78.20 ± 3.27	83.60 ± 2.70	80.80 ± 1.30	86.00 ± 3.81	87.00 ± 2.92
	KT+DE	✓	✓	×	74.00 ± 3.54	80.20 ± 3.83	85.80 ± 2.68	85.00 ± 2.35	89.40 ± 3.36	88.00 ± 2.00
	KT+FA	✓	×	✓	73.20 ± 2.77	78.60 ± 4.39	85.80 ± 3.56	82.40 ± 1.14	88.60 ± 3.85	88.60 ± 3.97
	PSA-AP	✓	✓	✓	**84.80 ± 4.02**	**86.60 ± 2.97**	**87.00 ± 3.61**	**88.60 ± 1.67**	**90.60 ± 2.41**	**90.60 ± 1.14**

## Data Availability

The audio data and source code are available from the corresponding author upon reasonable request. The data are not publicly available due to privacy and confidentiality.

## Abbreviations

The following abbreviations are used in this manuscript:

CNN	Convolutional neural network
EAT	Efficient Audio Transformer
MFCC	Mel-frequency cepstral coefficient
PSA-AP	Pig-sound spectrogram augmentation and angular-margin prototype alignment
SVM	Support vector machine
UAR	Unweighted average recall
